# Cocaine-Induced Steroid Resistant Organising Pneumonia in a Young Male: The Lows of Getting High

**DOI:** 10.7759/cureus.46923

**Published:** 2023-10-12

**Authors:** Zeenathalam Nadaf, Pratap Upadhya, Jeevanandham A, Sai Anudeep K, Madhusmita Mohanty Mohapatra

**Affiliations:** 1 Pulmonary Medicine, Jawaharlal Institute of Postgraduate Medical Education & Research, Puducherry, IND

**Keywords:** masson bodies, steroid resistant, organising pneumonia, crack cocaine, acute hypoxemic respiratory failure

## Abstract

Organizing pneumonia (OP) is a diffuse parenchymal lung disease occurring due to injury to the alveoli leading to typical histopathological features. Infections, connective tissue disorders, and medications are common aetiologies of OP. Cocaine-induced OP is uncommon. The patient had a fever and sore throat for two days corresponding to crack inhalation, followed by breathlessness that rapidly progressed to acute hypoxemic respiratory failure within one week. Radiology showed bilateral consolidation and ground glass opacities but did not respond to empiric treatment with antibiotics. After a multidisciplinary discussion, he was provisionally diagnosed as OP and treated with an intravenous methylprednisolone pulse dosage followed by oral prednisolone. OP was confirmed by surgical lung biopsy with the detection of Masson bodies. In view of progressive respiratory failure, steroid-resistant OP was diagnosed, and rituximab was administered as a second-line agent, but unfortunately, succumbed to respiratory failure. OP should be considered a differential in patients with consolidation who are non-responsive to initial conventional treatment. Multidisciplinary discussion and early lung biopsy to initiate immunosuppressants in the inflammatory stage of OP are emphasized for a possible better response.

## Introduction

Cocaine, commonly known as coca, is obtained from the leaves of Erythroxylon coca. Around 0.1% of the Indian population is estimated to abuse cocaine as per a household survey conducted by the United Nations Office on Drugs and Crime [1]. Ministry of Social Justice & Empowerment, India, in 2018 reported 9,40,000 cocaine users all over India [2]. Based on the route of use, cocaine has various pulmonary complications [3]. One such complication is cocaine-induced diffuse parenchymal lung disease-organizing pneumonia (OP), which usually responds to steroids and rarely leads to acute hypoxemic respiratory failure. We present a rare case of cocaine-induced rapidly progressive OP, which was resistant to systemic steroids, and discuss its course and possible best management.

## Case presentation

**Figure 1:**
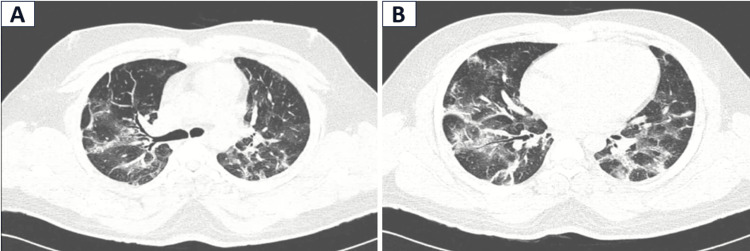
(A and B): HRCT Thorax showing ground glass opacities, airspace consolidation with air bronchograms, and interlobar septal thickening

We present a case of a 21-year-old male who was a current smoker with pack years of 0.8, with occasional cocaine use via smoking and snorting. Two weeks before he presented to us to emergency medical service (EMS), he had a history of fever and sore throat for two days. The fever was continuous with no associated chills or rigors, which lasted for two days and subsided on its own. It was associated with a sore throat. There was no cough, difficulty in swallowing, or change in voice. He continued to smoke and snort cocaine till one week after his symptom onset. Over the next two weeks, he developed acute onset progressive breathlessness, which worsened from Modified Medical Research Council (mMRC) grade zero to four, along with a dry cough. There was no history of hemoptysis. There was no significant weight loss or loss of appetite. There was no oral candidiasis/ skin lesions/ palpable peripheral lymphadenopathy to indicate immunocompromised status. He had fine end-inspiratory crepitations in bilateral infraaxillary and infrascapular areas on chest auscultation and a room air saturation of 90% and required 2L of oxygen by nasal prongs to maintain a saturation of 92-94%. On presentation, he was normotensive, tachypnoeic (30 breaths per minute), and tachycardic (110 beats per minute). Room air arterial blood gas analysis (ABG) was suggestive of type one respiratory failure (pH: 7.42, pCO2: 32.8 mm Hg, HCO3: 22.4 meq/liter, and pO2 of 58 mmHg), with a partial pressure of oxygen (pO2) to fraction of inspired oxygen (FiO2)- P/F ratio of 276 mmHg. Chest roentgenography showed bilateral reticular shadows, predominantly in bilateral lower zones. High-resolution computed tomography (HRCT) of the thorax showed bilateral patchy ground glass opacities (GGOs) and consolidation predominantly in lower lobes with a peri-broncho vascular distribution with interlobular septal thickening (Figures 1A, 1B). Total leukocyte counts were 8250 cells/microliter and hemoglobin of 16.9 g/dl. Renal and liver parameters were reported normal. Given the above clinical picture and radiology, the following diagnoses were considered and subsequently worked up: atypical pneumonia, viral pneumonia, bronchopneumonia, OP, pneumocystis jiroveci pneumonia, lymphoma, diffuse alveolar hemorrhage, nonspecific interstitial pneumonia (NSIP), pulmonary alveolar proteinosis (PAP), secondary to chronic myeloid leukemia (CML), bronchial mucosa-associated lymphoid tissue (MALToma). The patient was started on intravenous ceftriaxone and azithromycin empirically. A sputum biofire pneumonia panel was done, which was negative. Dengue (NS1 antigen and IgM ELISA), chikungunya real-time polymerase chain reaction (PCR), and scrub typhus real-time PCR were tested negative. Blood and sputum cultures for bacteria, Mycobacterium tuberculosis, and fungus were sterile. Procalcitonin was 0.05ng/ml. Peripheral blood smear and sputum cytology were done, suspecting PAP secondary to CML or Bronchial MALToma, but showed no abnormal cells/ blasts. Antinuclear antibody (ANA) and antineutrophil cytoplasmic antibodies (ANCA) tested negative. Serum benzoylecgonine was tested using liquid chromatography and mass spectrometry, which was positive for cocaine.

**Figure 2:**
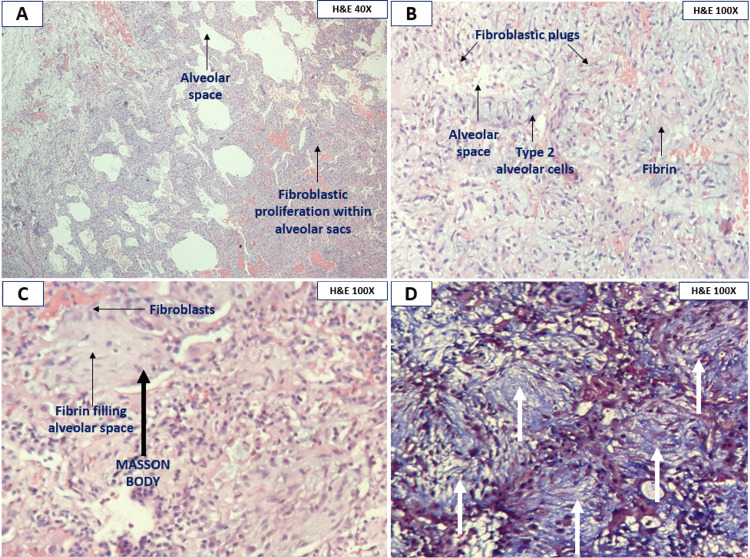
Histopathological examination of Surgical lung biopsy specimen A: Photomicrograph shows pleural lining, an excessive proliferation of fibrous tissue within the alveolar sacs. (H&E – Hematoxyline and eosin), 40X Low power. B: Photomicrograph shows organized plugs of intraluminal granulation tissue and Masson bodies. (H&E – Hematoxyline and eosin), 100X High power. C: Photomicrograph shows Masson bodies. (H&E – Hematoxyline and eosin), 100X High power. D: Photomicrograph shows Masson trichrome stain highlighting Masson bodies (white arrows). 100X High power.

**Figure 3:**
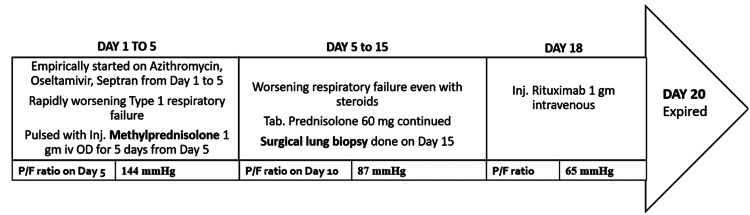
Schematic representation of the course of the patient

His respiratory failure worsened over the next five days with a 7 L oxygen requirement by facemask to maintain a saturation of 90%. His room air ABG showed a pO2 of 53 mmHg with a FiO2 of 50% (P/F ratio-108 mmHg). Intravenous cotrimoxazole was empirically started suspecting pneumocystis pneumonia but later stopped as induced sputum PCR was negative. Based on clinical characteristics and HRCT Thorax results, rapidly progressing cocaine-induced OP with acute hypoxemic respiratory failure was diagnosed following a multidisciplinary discussion (MDD) and a five-day course of antibiotics. On day five of admission, he was started on methylprednisolone 1 gm once daily by the intravenous route, which was continued for five days, followed by oral prednisolone 60 mg as a once-daily dosing. He did not improve despite steroids, with ABG showing a declining P/F ratio (88 mmHg). Hence it was decided to proceed with a surgical lung biopsy (SLB) from the left lower lobe on the 15th day of admission for a definitive diagnosis by histopathological examination (HPE). Spirometry could not be done because of respiratory failure. He belonged to the American Society of Anaesthesiologists (ASA) physical status classification class III with a risk of prolonged intubation, and likely so, the patient could not be extubated post SLB due to high respiratory rate (44 cycles per minute) on spontaneous mode of ventilation. He was sedated and paralyzed while on the pressure control mode of mechanical ventilation and was planned for tracheostomy. Meanwhile, the HPE of the biopsy specimen showed thickened alveolar septa lined by type II pneumocytes with fibroblastic plugs and Masson bodies within these alveoli, embedded with myxoid matrix admixed with inflammatory infiltrates in the interstitium. Masson's trichrome highlighted Masson's body sealing the diagnosis of OP (Figures 2A, 2B, 2C, 2D). In view of the worsening patient’s respiratory status (P/F ratio-67 mmHg), rituximab 1 gm was administered intravenously because of steroid-resistant disease on the 18th day of admission and was planned for a second dose after four weeks. His respiratory failure continued to worsen post Rituximab infusion with pO2 of 54.9 mmHg at a FiO2 of 100% and a P/F ratio of 55 mmHg. Despite maximal efforts, the patient gradually succumbed to the disease on the 20th day of admission. Figure 3 shows a schematic representation of the patient course. The patient was finally diagnosed with a case of cocaine-induced steroid-resistant rapidly progressive OP.

## Discussion

Cocaine is an alkaloid used in various forms like snorting, vaping, and intravenous injections. It interferes with the presynaptic reuptake of catecholamines and serotonin, giving rise to its sympathomimetic action, which leads to a central nervous system stimulant effect. Depending on the route, it has various pulmonary manifestations like crack lung, OP, diffuse alveolar hemorrhage, non-cardiogenic pulmonary edema, and interstitial fibrosis. OP is seen in free-base cocaine smokers (crack-smoking), the term crack coming from the sound that cocaine produces on heating [4]. OP is a diffuse parenchymal lung disease, histopathologically characterized by the presence of inflammatory cells and a connective tissue matrix within distal airspaces of the lungs. OP begins with the stage of "alveolar epithelial injury", which can be due to various reasons like infections, drugs, radiation, connective tissue disorders, aspiration, etc., which initiates migration and proliferation of fibroblasts and inflammatory cells towards the damaged epithelium with fibrin formation within the alveolar phase. The patient can clinically present with fever and dry cough at this stage. This is the stage where anti-inflammatory drugs and immunosuppressants are most effective. Further proliferation of fibroblasts and fibrin leads to the "alveolar organization phase", where alveolar spaces are filled with polypoid granulation tissue made of fibrin, fibroblasts, and inflammatory cells. At this stage, the patient clinically has breathlessness and a dry cough. The proliferation of granulation tissue buds filling the alveolar space (Masson bodies) is the key histological feature in OP. HRCT thorax shows multifocal areas of airspace consolidation, with air bronchograms and associated ground glass opacities, that are peripheral or peri-bronchial in distribution, lower lobe predominant, and tend to change over weeks. The final stage is the "resolution phase", where myofibroblasts proliferate, type 2 pneumocytes now line fibrin bundles, and epithelial continuity is maintained, leading to the resolution of symptoms [5].

**Figure 4:**
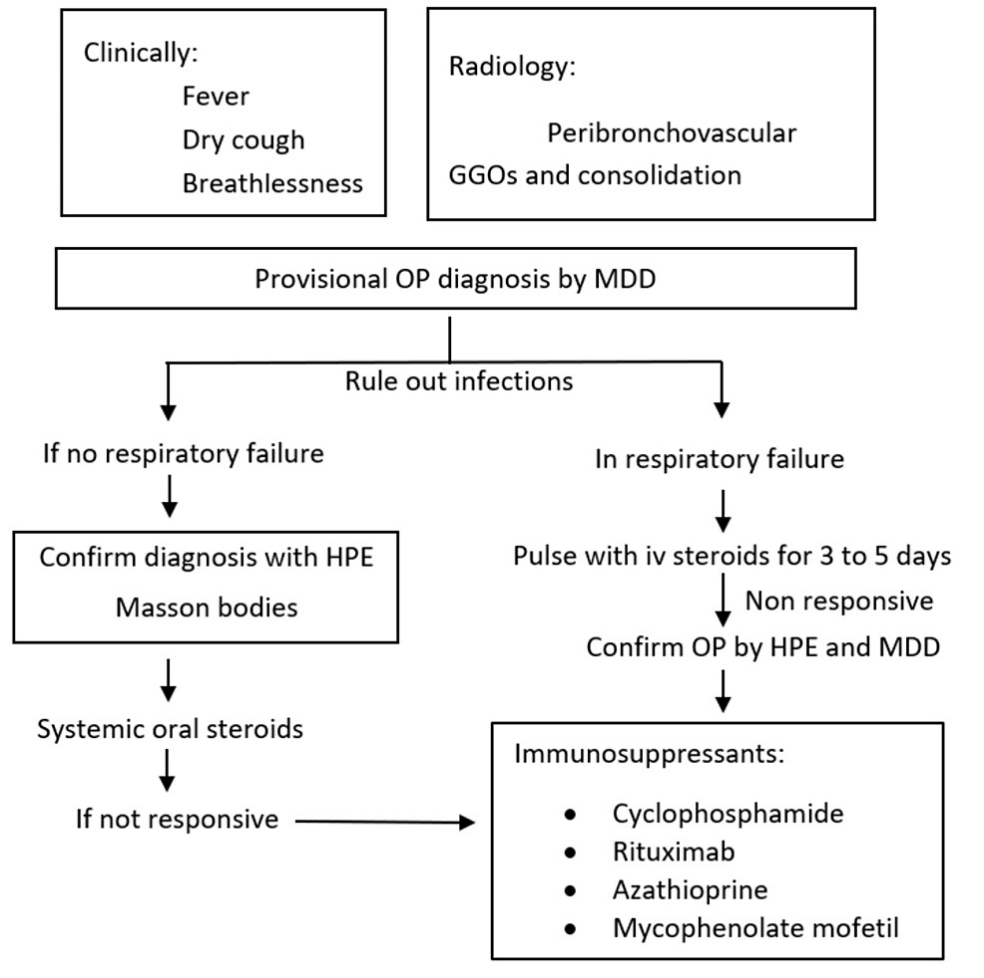
Proposed simplified flow chart for diagnosis and management of Organising pneumonia GGO: ground glass opacities, OP: organizing pneumonia, MDD: multidisciplinary discussion, HPE: histopathological examination)

Our patient had a previous history of fever and sore throat which corresponded to crack inhalation leading to epithelial injury, following which he developed acute onset breathlessness and cough. Respiratory failure further complicated the disease, with radiology corresponding to OP. As OP can have various aetiologies, cocaine-induced OP was diagnosed after ruling them out [6]. The definitive diagnosis of OP is by histopathological examination (HPE), and the mainstay of treatment is pulse steroid therapy [5]. For patients who present with rapidly progressive and extensive disease, causing respiratory failure, initial therapy with i/v glucocorticoids (e.g., methylprednisolone 125 to 250 mg every six hours or a pulse of 750 to 1000 mg once daily) is recommended to be given for three to five days [7]. However 40% of the patients with OP either do not respond to or stay dependent on steroids [8]. A second agent is added in such cases. Rituximab, azathioprine cyclophosphamide, cyclosporine A, mycophenolic acid, and intravenous immunoglobulins are recognized as possible second-line agents [8-11]. There is a paucity of data on the choice of agent for steroid-resistant OP. Based on previous case reports of successful therapy with Rituximab in the steroid-resistant OP, the same was administered [9,12]. A simplified flow chart proposed for the diagnosis and management of OP is shown in Figure 4.

The five-year survival rate is between 70% and 90 % in OP, and fatal outcomes are uncommon [13]. Unfortunately, our patient had progressive respiratory failure and succumbed to the disease.

## Conclusions

Cocaine use can cause various pulmonary complications, OP being one of them. OP is generally a chronic, nonfatal illness that responds to systemic steroids. Nevertheless, in some cases, it can present as rapidly progressive acute hypoxemic respiratory failure with steroid resistance. OP should be considered a differential in patients presenting radiologically with consolidation who are non-responsive to initial conventional treatment like antibiotics. MDD and early lung biopsy to initiate immunosuppressants in the inflammatory stage for possible better response is emphasised.
